# Persisting on Readability Could Provoke the Risk of Misinformation: A COVID-19 Pandemic Concern

**DOI:** 10.1017/dmp.2021.25

**Published:** 2021-02-08

**Authors:** Mohammad-Salar Hosseini, Mohammad Amin Akbarzadeh

**Affiliations:** 1 Student Research Committee, Tabriz University of Medical Sciences, Tabriz, Iran; 2 Research Center for Evidence-Based Medicine, Tabriz University of Medical Sciences, Tabriz, Iran; 3 Iranian Evidence-Based Medicine (EBM) Centre, Joanna Briggs Institute Affiliated Group, Tabriz, Iran

**Keywords:** comprehension, COVID-19, disease outbreak, readability, SARS-CoV-2

To the Editor,

We read the article by Basch et al. with great interest.^1^ As the novel idea of assessing the readability of publicly accessed information available on the Internet is appreciated, we wish to note some points regarding the mentioned research article. The necessity of general information being easy-to-read is indispensable, but we must agree that not all information is simplifiable. The unseen concern here lies in the fact that, while the general populace may not be capable of interpreting every kind of information in a correct and complete way, trying to deliver all information to the general audience may lead to misinterpretation, misjudgment, and misunderstanding. In other words, being uninformed about some questions may be much more harmless than provoking a misconception or misunderstanding among the population.^2-4^

In the study design, a crucial concern was raised regarding the search protocol. Some major websites, such as who.int, were excluded from the results because they are not included in the list of commercial websites (.com and.net), or noncommercial websites (.org,.gov, and.edu), and as we know, these websites play the most important role in informing the general population. The last concern to address is about the search terms, as we repeated the study design with 2 different keywords. Although the search results are definitely based on the location, using the keyword “COVID-19” instead of “Coronavirus”—the less correct and less common word for the purpose—led to remarkably different search results, which may strongly impact the outcomes of the research.

Moreover, we should consider the fact that a substantial proportion of society may not use the Internet and online tools to seek their desired information, chiefly relying on television and public broadcasting. This is especially evident in the less-educated portion of the society. Focusing on instructing the population about how to determine trustworthy references and distinguish reliable and unreliable sources of information may be a more reasonable and generally applicable approach toward dealing with possible comprehension flaws of the general population.
